# Vaccine antigens against fasciolosis in ruminants: a systematic review of antigen candidates, study characteristics, outcome measures, and reporting quality in cattle, sheep, and goats

**DOI:** 10.3389/fimmu.2026.1952671

**Published:** 2026-10-14

**Authors:** Guilherme Drescher, Larissa Americo, Tassia Cristina Bello de Vasconcelos, Vinícius Silva Belo, Michail Moroz, Fabiano Borges Figueiredo

**Affiliations:** 1 Cellular Biology Laboratory, Carlos Chagas Institute, Oswaldo Cruz Foundation (FIOCRUZ-PR), Curitiba, Brazil; 2 Laboratory of Parasitology and Parasitic Diseases (LAPAR-CAV-UDESC), Department of Veterinary Medicine, Agroveterinary Sciences Center, Santa Catarina State University, Lages, Brazil; 3 Auditora Fiscal Federal Agropecuária do Ministério da Agricultura Pecuária e Abastecimento, Brasília, Brazil; 4 Universidade Federal de São João Del Rei, Programa de Pós-graduação Ciências da Saúde, Divinópolis, Minas Gerais, Brazil; 5 Graduate Program in Animal Science, School of Medicine and Life Sciences, Pontifícia Universidade Católica do Paraná, Curitiba, Paraná, Brazil

**Keywords:** *F. hepatica*, *F. gigantica*, native and recombinant Antigens, systematic review, vaccine

## Abstract

The trematodes *Fasciola hepatica* and *Fasciola gigantica* exhibit a broad host range, infecting various mammalian species, including sheep, cattle, and, sporadically, humans. In cattle, fasciolosis constitutes a significant economic concern, often resulting in chronic and subclinical infections. Economic losses stem from liver condemnation and reduced weight gain in beef steers. Current disease control strategies include chemotherapy, livestock management practices, environmental measures to reduce intermediate host populations, and the development of vaccines for definitive hosts. Several antigens have been evaluated for their immunogenic potential in vaccine development against *Fasciola* species, including recombinant cathepsin 1 from *F. hepatica* (r*Fh*CL1) and recombinant fatty acid binding protein from *F. gigantica* (r*Fg*FABP), which have demonstrated promising immunogenic responses in cattle, sheep, and goats and can be candidates for a vaccine. It is important to thoroughly review the literature to identify studies that have tested various antigens in vaccine formulations against *F. hepatica* and *F. gigantica*, as this knowledge is crucial for advancing effective immunoprophylactic strategies. This systematic review identified the main antigen candidates, described the characteristics and outcome measures of the included studies, and assessed the reporting quality of studies evaluating vaccines against *F. hepatica* and *F. gigantica* in cattle, sheep, and goats. The review protocol was retrospectively registered on the Open Science Framework (OSF). The search employed multiple databases using defined keywords related to fasciolosis, immunization, and host species. Studies were screened and data extracted independently by two reviewers to minimize bias, with disagreements resolved by consensus with a third researcher. Methodological quality was appraised using the CONSORT checklist adapted from randomized controlled trials. The review included 37 studies evaluating vaccines against *F. hepatica* and 7 evaluating vaccines against *F. gigantica* in cattle, sheep, and goats. The most prevalent antigens included r*Fh*CL1 (10 studies for *F. hepatica*, of which four evaluated it as a single immunogen) and r*Fg*FABP (3 studies for *F. gigantica*). Overall, the included studies demonstrated suboptimal reporting quality. In conclusion, this systematic review identified r*Fh*CL1 and r*Fg*FABP as the most frequently evaluated antigens against *F. hepatica* and *F. gigantica*, respectively, across cattle, sheep and goats. Advancements in experimental models and improved study designs are imperative to clarify immune correlates of protection, optimize vaccine candidates, and ultimately enhance fasciolosis control in cattle, sheep and goats.

## 1 Introduction

*Fasciola hepatica* and *F. gigantica*, flukes responsible for fasciolosis, affect both domestic and wild animal populations (1, 2). Recognized as one of the Neglected Tropical Diseases (NTDs) by the World Health Organization (WHO), fascioliasis affects around 17 million people, with approximately 0.18 billion individuals at risk of infection (3). Fasciolosis, a highly prevalent neglected disease (4), causes substantial economic losses worldwide, estimated at approximately US$3 billion annually in the bovine livestock industry (5, 6), by reducing fertility, milk production, and weight gain, while also predisposing affected individuals to secondary infections (7–9). In cattle and small ruminants, *F. hepatica* infection causes hepatic damage through the migration of immature flukes within the liver parenchyma and the obstruction of the bile ducts by adult parasites. Consequently, livers affected by chronic infections are condemned due to the resultant pathology.

The pathogenesis of fascioliasis initiates with the accidental ingestion of encysted metacercaria by definitive hosts, which include humans and grazing animals (10). Upon reaching the small intestine, metacercaria undergo excystation, transforming into newly excysted juveniles (NEJs), which subsequently penetrate the duodenal wall (4). These NEJs migrate through the parenchyma, ultimately establishing themselves in the liver and gall bladder (1, 12). Once situated, they engage in sustained feeding, burrowing, and growth within the liver parenchyma. The management of fascioliasis primarily relies on anthelmintic treatments, with triclabendazole being a commonly used option. Presently, chemotherapy is commonly employed to mitigate clinical manifestations and minimize production losses associated with the disease (11). However, instances of resistance and treatment failures have been reported in various regions worldwide (13).

Addressing the challenges in controlling fascioliasis, including issues such as inaccurate diagnosis, drug resistance, and health risks to both humans and animals, necessitates the development of innovative diagnostic tools and novel immunotherapeutic control strategies, including vaccine (14, 15). Consequently, there is a pressing need to enhance our comprehension of the immune response to *F. hepatica*. Partial resistance to challenge infection with *F. hepatica* in rats and cattle has been observed, leading to reduced infection success (16). Conversely, in sheep, there is limited evidence indicating that primary sensitization decreases the worm burden resulting from secondary infection (17). Comparative analysis of the immune responses to secondary infection across these host species will contribute to elucidating protective immunity against *F. hepatica*.

Several mechanisms modulating humoral and cellular immune responses to primary *F. hepatica* infection have been elucidated (18, 19). *In vitro*, studies have demonstrated the release of cathepsin-B-like proteolytic enzymes by immature *F. hepatica*, which partially cleave immunoglobulins in various host species (18, 20, 21). Additionally, rapid turnover and excretion of the outer glycocalyx of juvenile flukes may prevent antibody-mediated eosinophil attachment (18, 22). *In vivo* experiments have indicated a decrease in the proliferative response of sheep lymphocytes to mitogens during fascioliasis, suggesting the development of immunodepression (18, 23).

The antigen-specific immune response to primary *F. hepatica* infection has also been investigated and can be used in fascioliasis vaccine (2, 24). While the humoral response has been extensively studied in various hosts, specific immunoglobulin isotypes have been characterized only in rats and humans (18). Additionally, early and transient antigen-specific lymphocyte responses have been observed in rats and cattle post-experimental infection. The colonization of the host by the parasite is favored by its protease enzymes, which fulfill several vital functions such as tissue invasion, parasite nutrition, and evasion of the host immune response (18). Understanding the primary antigens tested in vaccines against *F. hepatica* is crucial for developing effective immunization strategies. Knowledge of these key antigens allows for targeted vaccine design, improving protective responses and controlling fasciolosis more efficiently.

The available evidence remains fragmented, and no comprehensive synthesis has characterized the antigen candidates evaluated across ruminant hosts, the methodological features and outcome measures of these studies, and the completeness of their reporting. Addressing this gap is important for guiding future research and supporting the development of vaccines against fasciolosis. We therefore conducted a systematic review to identify the main antigen candidates, describe the characteristics and outcome measures of the included studies, and assess the reporting quality of studies evaluating vaccines against *F. hepatica* and *F. gigantica* in cattle, buffaloes, sheep, and goats. By providing an overview of the existing evidence and its reporting limitations, this review identifies relevant knowledge gaps and priorities for future vaccine research.

## 2 Methods

### 2.1 Identification and selection of studies

A systematic search was conducted in MEDLINE (via PubMed), Google Scholar, and the Cochrane Library (via the Virtual Health Library) to identify studies evaluating antigen candidates for vaccines against fasciolosis in cattle, buffaloes, sheep, and goats. Because this systematic review analyzed previously published studies, ethical approval and informed consent were not required. The protocol for this systematic review was registered as retrospective in the Open Science Framework (OSF) on July 8, 2026 (https://osf.io/mwgvf; DOI: 10.17605/OSF.IO/39WHS), and the review was conducted in accordance with the PRISMA guidelines 2020 (25). The completed PRISMA 2020 checklist was added as **Supplementary Table 4**.

The initial searches of MEDLINE (via PubMed), Google Scholar, and the Cochrane Library were conducted in June 2024 and updated on May 10, 2026; all three databases were last searched on the same date. The search strategy with index terms and the inclusion and exclusion criteria are presented in **Table 1**. The Rayyan systematic review platform (26), was used to manage the screening process and remove duplicate records and reference lists of retrieved publications were also hand-searched for additional studies.

**Table 1:** Search strategies and inclusion and exclusion criteria applied in the systematic review of peptides with vaccine potential against *F. hepatica* and *F. gigantica* in cattle, goats, and sheep.

**Search strategy:** **PUBMED:** (Fasciola OR Fasciola hepatica OR Fasciola gigantica OR Fasciolosis OR Fascioliasis) AND (Ruminant OR Cattle OR Bos OR Bovine OR Sheep OR Ovis OR Goats OR Buffaloes OR buffalo) AND (vaccination OR immunization OR antibody response OR cellular Immunity OR Immunotherapy) **Google Scholar:** (Fasciola OR Fasciolosis OR Fascioliasis) (Ruminant OR Cattle OR Bos OR Bovine OR Buffaloes OR Sheep OR Ovis) (vaccination OR immunization OR antibody response OR humoral response OR cellular response OR prevention OR Immunotherapy) **COCHRANE LIBRARY:** MeSH descriptor: (Fasciolosis OR Fascioliasis) AND (Vaccines OR Vaccination)
**Inclusion criteria:** Inclusion was based on the following criteria: a) original articles published in English; b) studies evaluating the efficacy of vaccines against *Fasciola hepatica* and *Fasciola gigantica* infection; c) using placebo or other vaccines as a control; d) including cattle, buffaloes, goat, and sheep, with no age restriction or previous contact with the parasite, and with immune response as the outcome.
**Exclusion criteria** Publications covering the following topics were excluded: 1) studies in others animal species; 2) editorials, letters, review articles and conference abstracts; 3) studies in *Fasciola magna*;

### 2.2 Data extraction

Data extraction was performed systematically from eligible studies to capture key methodological, immunological, and efficacy-related variables essential for evaluating vaccine candidate antigens against *F. hepatica* and *F. gigantica*. GD and LA, followed fixed protocol to extract data from the eligible studies: (1) vaccine antigen; (2) antigen concentration; (3) vaccine adjuvant; (4) vaccine doses; (5) vaccine intervals; (6) infectant doses and others important information about the studies. Additional information were requested from the authors of included studies when necessary. Any disagreements between reviewers were resolved through consultation with a third researcher to ensure consensus and accuracy. This review focused exclusively on studies available as full-text articles, thereby excluding abstracts, conference proceedings, and unpublished data, which may introduce the possibility of publication bias.

#### 2.2.1 Journal impact factor

The studies were divided into three categories: impact factors less than 10, impact factors greater than 10 but less than 20, and impact factors greater than 20. The journal impact factors applied to all selected articles were those reported for the year 2025. A journal impact factor of 10 and above is considered high in most study fields (27). JIF was accessed in through the Carlos Chagas Institute – ICC access to the Journal Citation Reports on the website: https://jcr.clarivate.com.

### 2.3 Reporting quality assessment

Methodological quality was assessed independently by two reviewers (Guilherme Drescher and Larissa Américo – GD and LA) using the CONSORT checklist, which was adapted for the evaluation of randomized controlled clinical trials (28, 29). It is important to note that the CONSORT instrument primarily assesses the completeness and transparency of trial reporting rather than methodological rigor per se; accordingly, it was employed in this review solely to evaluate reporting quality. Prior to full data extraction, the two reviewers piloted the checklist on a randomly selected subset of studies, encompassing both *F. hepatica* and *F. gigantica* vaccine trials. During this pilot phase, the reviewers discussed each CONSORT item, its operational definition, and the criteria for classifying items as “reported,” “unclear,” or “not reported,” in accordance with the official CONSORT explanatory document. Each item was subsequently scored as 1 if adequately reported and 0 if not reported or ambiguously described. Items referenced in the trial’s **Supplementary Materials** were considered reported only if explicitly cited in the main article. For each study, the overall reporting quality was quantified as the proportion of applicable CONSORT items fulfilled, calculated by dividing the number of reported items by the total number of applicable items. In cases where the two primary reviewers disagreed on any scoring decision, a third independent reviewer was consulted, and the final classification was reached through consensus discussion.

## 3 Results

### 3.1 Study search and selection

The initial search yielded 751 records from PubMed, 5,000 articles from Google Scholar, and no records from the Cochrane Library. After title screening, 5,599 records were excluded, leaving 152 studies that were considered potentially eligible for inclusion. These records were then analyzed according to the predefined search strategy, and after the removal of duplicate entries (n = 20), 132 titles remained. Of these, 18 were excluded following abstract reading, resulting in 114 papers for full-text assessment. Among these, 10 were discarded as review articles, five were excluded for not being published in English, and a further 27 were excluded because they addressed immune responses to infection rather than vaccine efficacy. Consequently, 72 full-text articles were assessed for eligibility. Of these, 16 were excluded due to the unavailability of humoral response data, eight were removed for involving non-target animal species in *F. hepatica* studies, and four were excluded for evaluating *F. gigantica* immunity in non-ruminant species. Ultimately, 44 studies were included in the qualitative synthesis, comprising 37 studies on *F. hepatica* and 7 studies on *F. gigantica* (**Figure 1**). Notably, none of the included studies simultaneously evaluated vaccines against both *F. hepatica* and *F. gigantica* within the same experimental trial, underscoring a significant gap in comparative vaccine research across these two economically important trematode species.

**Figure 1:**
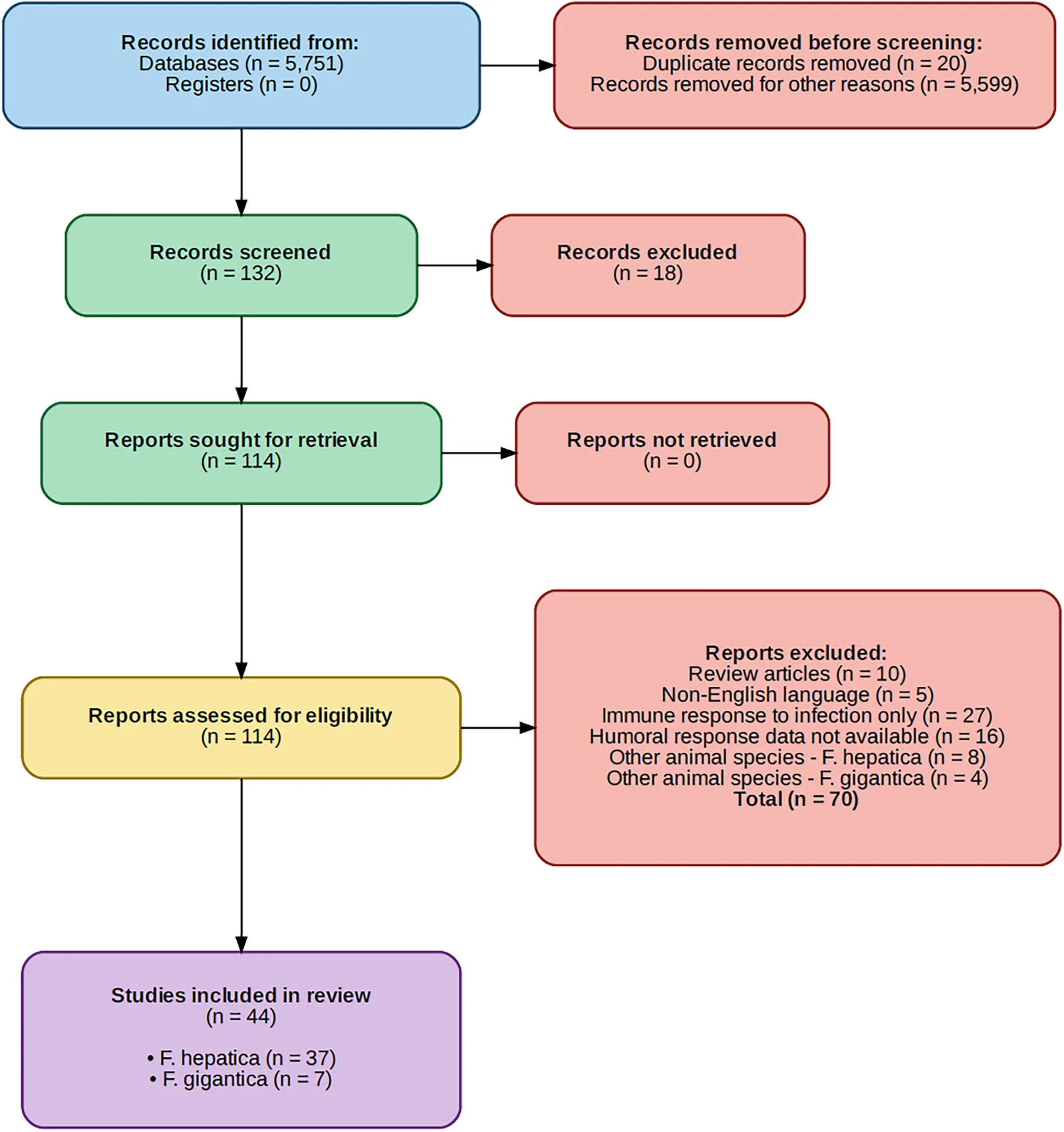
Flow diagram of the study selection process (25).

### 3.2 Vaccine antigens

Different antigens have been investigated over the years in the development of vaccines against *F. hepatica* and *F. gigantica*, with some of the earlier approaches relying on whole-parasite preparations. Notably, Younis et al. (1986) (30) and Creaney et al. (1995) (31) conducted studies employing irradiated *Fasciola* species, specifically *F. gigantica* and *F. hepatica*, respectively. Younis et al. (30), administered irradiated *F. gigantica* orally to bovines and evaluated fluke burden as the primary outcome. Highly irradiated parasites stimulated more resistance than those less irradiated; however, there was no significant difference in the parasite count in vaccinated and non-vaccinated animals. In contrast, Creaney et al. (31) orally challenged sheep with irradiated *F. hepatica* and assessed a broader spectrum of parameters, including serology, fluke burden, fecal egg count, macroscopic liver damage, and liver enzyme assays. Despite a comprehensive evaluation, Creaney et al. (31) found no significant reduction in egg shedding nor observable differences in liver pathology between vaccinated and control groups.

#### 3.2.1 F. hepatica

In the studies included in this systematic review investigating *F. hepatica*, a diverse array of protein antigens was utilized, encompassing both native and recombinant forms, with ten distinct antigen types identified (**Supplementary Table 2**). The r*Fh*CL1 was the most frequently employed antigen, featured in ten studies. Additionally, unique approaches were observed: one study evaluated the efficacy of the Foot-and-Mouth Disease Virus (FMDV) vaccine against *F. hepatica* in cattle, while two studies explored bacteriophage-based interventions. Furthermore, two investigations assessed the protective effects of a recombinant antigen derived from *Schistosoma mansoni* fatty acid-binding protein 14 (r*Sm*14) in goats and sheep. Notably, one article used irradiated *F. hepatica* as the immunogen (31).

In the evaluation of *F. hepatica* vaccine studies, methodological approaches to confirming the parasite-free status of animals before antigen inoculation displayed notable variability. Specifically, five studies (32–36) did not employ any diagnostic method to verify the absence of infection in the experimental subjects. Conversely, four studies implemented anthelmintic treatment regimens to clear existing parasitic infections before initiating vaccination trials, thereby aiming to standardize baseline infection status. Additionally, most of the remaining investigations relied on serological analysis via ELISA and fecal egg counts, either independently or in combination, to classify animals as negative for *F. hepatica*, ensuring controlled experimental conditions.

In the evaluated studies targeting *F. hepatica*, antigen concentrations demonstrated notable variability, ranging from 100 µg to 500 µg per dose. The predominant route of vaccine administration was subcutaneous, reflecting its widespread acceptance in veterinary immunization protocols. A particularly multivalent vaccine strategy was described in the study by Cwiklinski et al. (2023) (37). This involved the combination of up to four distinct recombinant antigens (recombinant antigen peroxiredoxin (r*Fh*Prx), recombinant antigen thioredoxin (r*Fh*Trx), recombinant thioredoxin-glutathione reductase (r*Fh*TGR), recombinant superoxide dismutases (r*Fh*SOD1 and r*Fh*SOD3) per inoculation, with each antigen administered at a minimum concentration of 25 µg.

Moreover, five studies incorporated bacteriophage-based vaccine platforms to elicit humoral immunity against *F. hepatica*. In these cases, the phage particles were administered at a high dose of 1 × 10^13^ particles per inoculation. In the analysis of vaccine formulations against *F. hepatica*, the systematic review identified considerable variability in the use of adjuvants across 37 studies. Notably, three studies did not employ any adjuvant, while only one utilized an oil-based adjuvant. In contrast, most of the remaining studies incorporated a diverse array of over five different adjuvant types, reflecting the experimental exploration of various immunostimulatory strategies. Among these, Freund’s Complete Adjuvant (FCA) and Freund’s Incomplete Adjuvant (FIA) were the most prevalently used, featuring prominently in eight distinct studies (17, 32, 38–42).

In the evaluation of vaccine efficacy against *F. hepatica*, a diverse array of 15 different assessment methods was identified across thirty-seven studies selected, underscoring the complexity and multifaceted nature of vaccine impact measurement in fasciolosis research. Serology, fluke burden, eosinophil count, liver damage and duodenal damage, lymphocyte proliferation assays, weight gain, fecal egg counts, wet weight fluke, size of fluke, gross pathological evaluation, antibody avidity, histopathology, cytokine production and peritoneal cells population were the assessment methods identified in this systematic review for *F. hepatica*. Notably, serological assessment via ELISA was the predominant method, utilized in 35 of the 37 studies, highlighting its role as a reliable and sensitive tool for quantifying immunological responses following vaccination. Exceptions to this trend include the studies (22, 34), which did not employ ELISA serology and utilized other methodologies for vaccine evaluation.

Beyond serology, parasitological parameters such as fluke burden and egg counts in feces and bile were the next most frequently applied outcome measures, providing direct evidence of parasitic load reduction as an indicator of vaccine-induced protection. Additional evaluations commonly reported included liver damage, assessed either macroscopically or microscopically, and weight gain in kilograms, which serve as critical indicators of the physiological and pathological impact of infection and the protective benefit conferred by vaccination.

#### 3.2.2 F. gigantica

In studies involving *F. gigantica*, the r*Fg*FABP emerged as the most frequently used antigen, being employed in three out of seven analyzed articles (**Supplementary Table 1**). Notably, one of these three studies (45), combined r*Fg*FABP with native antigens, exploring a multivalent approach. Additionally, two other studies exclusively utilized native antigens, and among them, one investigated irradiated *F. gigantica*. In studies evaluating *F. gigantica* vaccine efficacy, two articles employed both serological testing and fecal egg counts as diagnostic tools to confirm the absence of parasitic infection in the experimental animals. Additionally, two investigations incorporated anthelmintic treatment protocols to eliminate existing infections before commencing immunization trials. However, it is noteworthy that Mahana et al. (2016) (43), did not provide explicit details regarding the preparation and pre-experimental management of the inoculated animals.

The concentrations of antigens used for vaccine doses in the evaluated in *F. gigantica* studies ranged broadly from 25 µg to 500 µg, with the subcutaneous route being the most employed method of administration. In studies focusing on *F. gigantica*, different adjuvants were utilized to enhance immune response, including Freund’s Complete Adjuvant (FCA) and Freund’s Incomplete Adjuvant (FIA), which were applied in two of the articles (43, 46). The interval between vaccine doses varied between 2 and 5 weeks, reflecting some heterogeneity in immunization protocols.

In the assessment of vaccine efficacy against *F. gigantica*, eleven distinct evaluation methods were identified across the seven included studies, reflecting a multifaceted approach to measuring immune protection. Serology, worm wet weight, worm length/width, scanning electron microscopy (SEM) of tegument, hepatic lesion scoring, fluke burden, eosinophil count, liver enzyme assays, lymphocyte proliferation, size of fluke and fecal egg count were the assessment methods identified in this systematic review for *F. gigantica.* Among these, serological analysis through ELISA was the predominant technique, employed in six of the seven studies, underscoring its critical role in quantifying antibody-mediated immune responses post-vaccination. Additionally, parasitological metrics such as fluke burden and egg counts in feces and bile constituted the next most frequently utilized outcome measures, serving as direct indicators of infection intensity and vaccine-induced parasite reduction. Notably, Nambi et al., (46), uniquely evaluated fluke size as an efficacy parameter, comparing vaccinated groups to controls, thereby introducing an innovative dimension for understanding vaccine impact on parasite development.

### 3.3 Location of the studies and the ethical committee approval

In the present systematic review, a total of 44 studies evaluated *Fasciola* vaccine candidates. Of the 37 were *F. hepatica* where 22 studies did not report on the geographic location where the research was conducted, whereas only 15 provided such information (**Supplementary Table 2**). Among the studies that specified location, the geographic distribution was as follows: two were conducted in Ireland using sheep, one in Ireland using cattle, four in Spain using sheep, two in Mexico using sheep, one in Mexico using goats, one in the United Kingdom using cattle, one in Australia using sheep, one in Switzerland using cattle, one in Uruguay using sheep and one in Uruguay using cattle. In contrast, seven studies focused on *F. gigantica* (**Supplementary Table 1**). These comprised two investigations from India involving buffaloes, and one study each from Australia (cattle), Iran (sheep), Sudan (cattle), Egypt (sheep), and Thailand (goat) (**Figure 2**).

**Figure 2:**
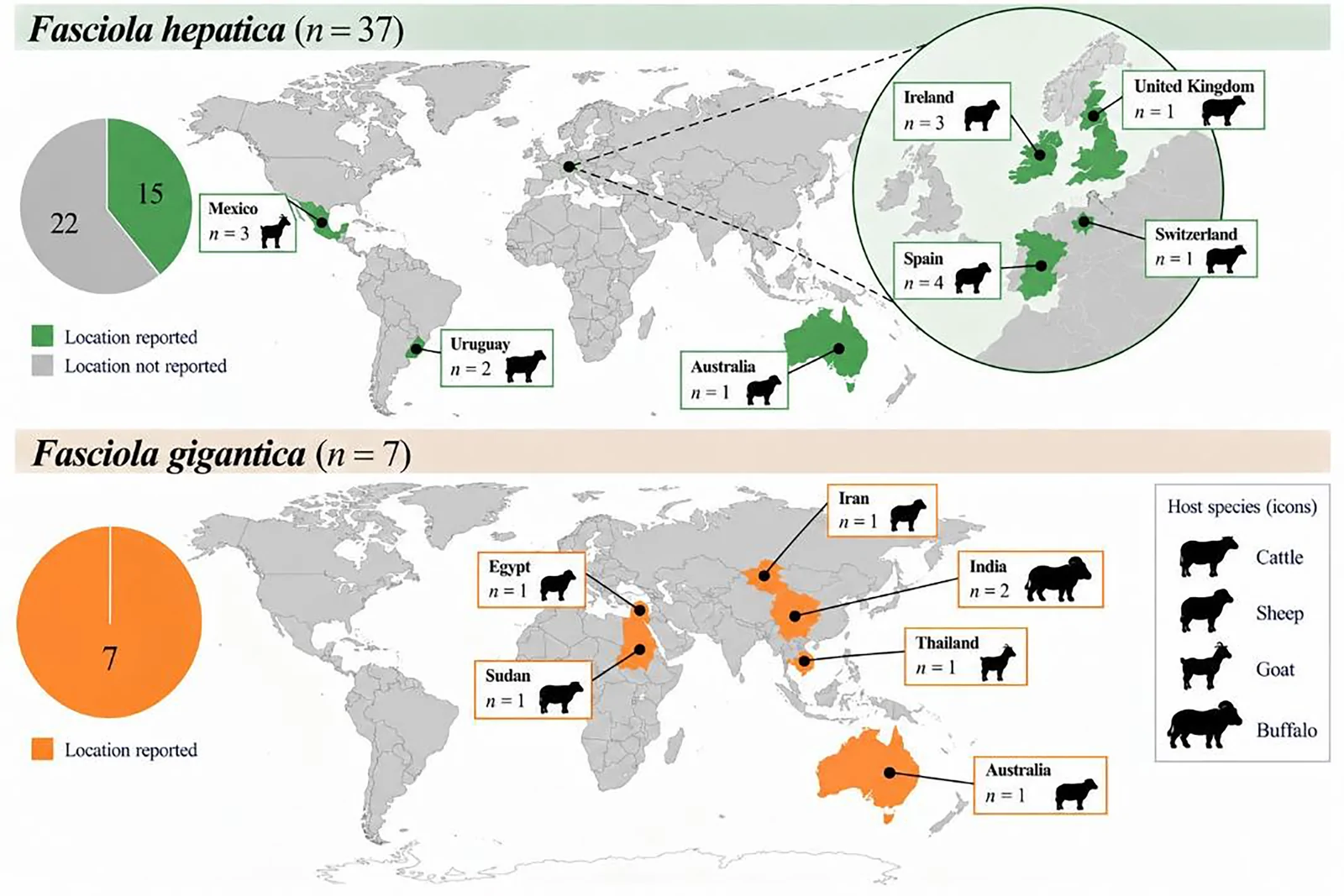
Geographic distribution of studies evaluating *Fasciola* vaccine candidates.

Regarding ethical oversight, 12 (32.4%) of the 37 *F. hepatica* studies did not report approval from an ethics committee, seven of which were published before 2000. Among the seven *F. gigantica* studies, only two—Mahana et al. (2016) (43) and Sangfuang et al. (2025) (47)—explicitly reported ethical approval in the manuscript. Of the remaining studies, three were published after 2000 (46, 48, 56). Two were conducted before 2000; Estuningsih et al. (1997) (45) and Younis et al. (1986) (30).

### 3.4 Journal impact factor’s

The JIFs in which these studies were published varied considerably. Notably, none of the articles identified in this systematic review were published in journals with an impact factor exceeding 5. For *F. hepatica* studies, the journal impact factors ranged from 0.8 to 4.5, with most articles appearing in journals possessing an impact factor between 1.5 and 2.5 (**Supplementary Table 2**). For *F. gigantica* studies, the journal impact factors ranged from 1.3 to 3.7, comprising one article with an impact factor of 1.3, two articles with an impact factor of 1.8, and the remaining articles published in journals with impact factors above 2.0. It is important to note that for both *F. hepatica* and *F. gigantica* studies, the reported impact factors correspond to the year 2025 (**Supplementary Table 1**). This distribution highlights the range of publication venues utilized by researchers in the field, from more specialized journals to those with broader scientific reach.

### 3.5 Justification of the sample size and justification of the study

None of the selected studies, whether on *F. hepatica* or on *F. gigantica*, justified the sample size used in their research, indicating a notable gap in methodological rigor related to study design. Regarding the scientific background justification, a disparity was observed between species: for *F. hepatica*, three studies lacked an adequate rationale supporting the investigation within their scientific context, whereas all studies concerning *F. gigantica* presented a well-articulated and robust justification for the relevance and necessity of the research.

### 3.6 Overall quality of reporting

#### 3.6.1 F. gigantica

The methodological quality assessment of studies evaluating vaccine candidates against *F. gigantica* demonstrated substantial deficiencies in compliance with the CONSORT 2010 guidelines (**Supplementary Table 3**). None of the seven included studies fulfilled all 37 items of the CONSORT checklist. The highest level of compliance was observed in three studies Nambi et al. (2005) (46), Sangfuang et al. (2025) (47) and Paykari et al. (2002) (48), which reported 13 of the 37 checklist items (35.1%), whereas the remaining studies fulfilled only 11 or 12 items (29.7–32.4%). None of the publications identified the study as a randomized trial in the title or provided a structured abstract according to CONSORT recommendations. Moreover, essential methodological information—including sample size calculation, allocation concealment, blinding procedures, trial registration, protocol availability, and baseline demographic or clinical characteristics of the experimental groups—was consistently absent across all studies, highlighting important limitations in the reporting quality of *F. gigantica* vaccine trials.

#### 3.6.2 F. hepatica

The methodological quality assessment of studies evaluating vaccine candidates against *F. hepatica* revealed substantial deficiencies in compliance with the CONSORT 2010 guidelines (**Supplementary Table 3**). None of the 37 included studies fulfilled all 37 items of the CONSORT checklist. The highest level of compliance was observed in twelve studies, each fulfilling only 9 of the 37 checklist items (24.3%), while a further fourteen studies met only 8 items (21.6%), indicating consistently poor reporting quality across the literature. None of the publications identified the study as a randomized trial in the title or provided a structured abstract in accordance with CONSORT recommendations. Moreover, essential methodological information—including sample size calculation, allocation concealment, blinding procedures, trial registration, protocol availability, and comprehensive baseline demographic or clinical characteristics of the experimental groups—was consistently absent. At the lower end of reporting quality, the study by Morrison et al. (1996) (49), fulfilled only 4 of the 37 CONSORT checklist items (10.8%), illustrating the historical limitations in the reporting standards of *F. hepatica* vaccine trials.

## 4 Discussion

To our knowledge, this systematic review represents a comprehensive evaluation of vaccine candidate antigens against *F. hepatica* and *F. gigantica* across multiple host species, including cattle, sheep, and goats. A total of 44 studies were included in the qualitative synthesis, with 37 targeting *F. hepatica* and 7 focusing on *F. gigantica*, encompassing a diverse geographic distribution and variable methodological rigour. Despite variability in study design, antigen selection, and immunization strategies, 94.6% (35/37) of the studies utilized standardized immunological assessments such as ELISA to gauge humoral responses, while parasitological evaluations often employed fecal egg counts as a diagnostic benchmark.

The *F. hepatica* r*Fh*CL1 is recognized as one of the most abundantly expressed and immunologically significant proteins secreted by the parasite, playing a central role in host immune modulation. This characteristic makes r*Fh*CL1 a prominent candidate antigen for the development of vaccines targeting fasciolosis. In our review, we identified four studies specifically evaluating the efficacy of r*Fh*CL1 as a single immunogen in goats, sheep, and cattle (44, 50–52). The evaluated studies included r*Fh*CL1 as the most frequently tested antigen, along with a range of other antigens, including additional cathepsins (53–55). For the *F. gigantica* recombinant proteins, such as r*Fg*FABP, in vaccine development, while also maintaining interest in native antigen preparations as potential immunogens (46, 56).

The heterogeneity of the antigen selection reflects the ongoing search for effective vaccine candidates, leveraging both homologous and heterologous antigen sources as well as innovative immunization strategies against *F. hepatica* infection. The comparative evaluation of native and recombinant antigens as vaccine candidates has been a recurring strategy in the quest to develop effective immunizations against both *F. hepatica* and *F. gigantica*. Notably, studies such as those by Orbegozo-Medina et al. (2018) (57) and Estuningsih et al. (1997) (45), have undertaken this approach for two parasites. The use of phage-display technology as an immunization strategy highlights emerging trends in vaccine development, leveraging viral vectors to present parasite antigens efficiently and stimulate good antibody responses (58–61).

The variation in journal impact factors among studies on *F. hepatica* and *F. gigantica* vaccines reveals important information about the dissemination and academic reach of research within this field. For *F. hepatica*, the predominance of publications in journals with moderate impact factors, mostly between 1.5 and 2.5, suggests that while this research is gaining recognition, it is still largely concentrated within specialized parasitology or veterinary science outlets rather than broad, high-impact multidisciplinary journals (33, 35, 41, 61–64). Similarly, studies on *F. gigantica* appeared in journals ranging from 1.3 to 3.7 in impact factors, indicating a very similar impact factor in the articles selected (48, 56).

The diverse experimental strategies employed in *F. hepatica* vaccine studies reflect essential efforts to maintain controlled and reproducible conditions. Research on large animals (sheep, cattle, goats and buffaloes) is very complicated for animal pathogen free acquisition (17, 39, 65, 66). The heterogeneity observed in pre-experimental animal screening and management protocols underscores a critical challenge within *F. gigantica* vaccine research, as variations in baseline infection status and animal preparation may significantly influence immunological outcomes and hinder direct comparability across studies (48, 56). Furthermore, the wide-ranging approaches in antigen dosage, adjuvant use, and immunization schedules demonstrate the ongoing endeavor to identify optimal combinations that maximize protective immunity (45, 60, 67). Notably, the adoption of combinatorial vaccine designs, which involve multiple antigens targeting distinct parasite epitopes, represents a promising advancement that could enhance the breadth and robustness of immune responses against fasciolosis (50–52, 68, 69). Collectively, these methodological variations highlight both the complexity of vaccine development against *Fasciola* species and the necessity for standardized protocols to improve reproducibility and facilitate translational progress in this neglected tropical disease.

The overall methodological quality of the included studies was generally poor according to CONSORT guidelines, with frequent deficiencies in sample size justification, randomization, and blinding procedures (58, 70). Nonetheless, this review highlights significant advances in the understanding of immunotherapeutic approaches to fasciolosis and underscores the urgent need for improved reporting standards to enhance reproducibility and rigour in future vaccine research within this field (32, 38). Collectively, these findings demonstrate a pressing need for more stringent adherence to standardized reporting protocols to enhance methodological transparency, facilitate critical appraisal, and ultimately improve the reliability of vaccine efficacy assessments. Addressing these gaps will be crucial for advancing the development of effective *F. hepatica* vaccines and fostering comparability across future clinical investigations.

The innovations of this systematic review include: (i) the first comprehensive synthesis of vaccine antigens tested against both *F. hepatica* and *F. gigantica* across cattle, sheep, and goats; (ii) the CONSORT checklist was applied to all 44 included studies (37 targeting *F. hepatica* and 7 targeting *F. gigantica*); (iii) Geographic location was underreported in 22 of the 37 *F. hepatica studies* (59%) and in none of the *F. gigantica* studies; and (iv) the provision of a detailed overview of antigen concentrations, adjuvant formulations, and administration routes employed in the field.

These findings provide several actionable insights for future research. First, standardized protocols for pre-experimental screening, sample size calculation, randomization, and blinding should be adopted to enhance methodological rigor and reproducibility. Second, the exploration of multivalent vaccine formulations, combining multiple antigens targeting distinct parasite epitopes, represents a promising avenue for improving protective efficacy. Third, direct comparative trials evaluating vaccine candidates against both *F. hepatica* and *F. gigantica* within the same experimental setting are urgently needed to identify cross-protective antigens. Fourth, mandatory reporting of geographic location and adherence to CONSORT guidelines should be enforced by journal editors to improve transparency and facilitate meta-analytic synthesis. Finally, future investigations should prioritize the optimization of adjuvant-antigen combinations through head-to-head comparative studies to maximize humoral and cellular immune responses across different host species.

The review does not synthesize or interpret the efficacy outcomes of the included studies, focusing strictly on reporting parameters. Nonetheless, the gaps and deficiencies identified herein underscore the pressing need for improved study design, standardized methodologies, and enhanced reporting practices to advance the development of effective vaccines against fasciolosis in ruminants.

### 4.1 Strengths and limitations

A systematic search strategy was employed to comprehensively identify all original vaccine efficacy studies targeting *F. hepatica* and *F. gigantica* infections in relevant hosts, including cattle, sheep, and goats. To reduce potential selection bias, two independent reviewers conducted the study screening and data extraction processes. Exclusion criteria encompassed studies on non-target species, editorials, reviews, conference abstracts, and investigations involving *F. magna*.

Regarding the verification of prior exposure to *Fasciola* infection, considerable heterogeneity was observed across the included studies. While some investigations employed serological assays (ELISA) and fecal egg counts to confirm the parasite-free status of animals prior to vaccination, others implemented anthelmintic treatment regimens to clear existing infections, and a subset of studies did not report any diagnostic confirmation of pre-experimental infection status. This lack of standardization represents a notable methodological limitation that may influence the interpretability and comparability of vaccine efficacy outcomes.

The data extraction and quality assessment process applied equal weighting to all items adapted from relevant reporting guidelines pertinent to vaccine trials. It is acknowledged, however, that certain elements, such as detailed methodological descriptions, may bear greater importance in evaluating the rigor and transparency of the studies. Consequently, studies achieving comparable overall scores may differ in the comprehensiveness of their methodological reporting. Importantly, this systematic review evaluated the quality and completeness of reporting in *Fasciola* vaccine studies rather than appraising their methodological robustness or risk of bias. Therefore, a study with inherent bias but thorough reporting was credited fully for reporting quality alone. A formal risk-of-bias assessment using established tools, such as the Cochrane Risk of Bias tool or ROBINS-I, was not performed, as this was beyond the scope of the present review.

## 5 Conclusion

Our analysis revealed that the quality of reporting in *Fasciola* vaccine efficacy studies was generally suboptimal. This finding should not be interpreted as diminishing the scientific value of the existing body of evidence. Rather, it highlights the continued need for greater transparency and rigor in the reporting of vaccine trials within the *Fasciola* research community. This is particularly important because ongoing and future vaccine development studies rely on the foundational data provided by previous research. In light of these findings, we advocate that journal editors enforce stricter reporting requirements, including the mandatory submission of a completed CONSORT checklist alongside manuscripts, to improve reporting completeness. We also encourage authors to provide more detailed descriptions of their methods, particularly key aspects such as sample size calculation, randomization procedures, and blinding methods. These improvements will promote greater reproducibility, comparability, and confidence in *Fasciola* vaccine research findings.

## Data Availability

The original contributions presented in the study are included in the article/**Supplementary Material**. Further inquiries can be directed to the corresponding authors.
