# Supplementary material for: Vaccine antigens against fasciolosis in ruminants: a systematic review of antigen candidates, study characteristics, outcome measures, and reporting quality in cattle, sheep, and goats
Source: Front Immunol. 2026 Oct 14;17:1952671. doi: 10.3389/fimmu.2026.1952671 (PMC13713426; doi:10.3389/fimmu.2026.1952671)
Supplement: Supplementary file 1 [file DataSheet1.pdf]

PRISMA 2020 Checklist

Section and Topic	Item #	Checklist item	Location where item is reported
**TITLE**	**TITLE**	**TITLE**	**TITLE**
Title	**1**	Identify the report as a systematic review.	Title page. The title explicitly contains the phrase “A Systematic Review”.
**ABSTRACT**	**ABSTRACT**	**ABSTRACT**	**ABSTRACT**
Abstract	**2**	See the PRISMA 2020 for Abstracts checklist.	Abstract. Structured abstract reports background, objectives, methods (databases, dual independent screening, CONSORT appraisal), main results (principal antigens and study numbers), and conclusions.
**INTRODUCTION**	**INTRODUCTION**	**INTRODUCTION**	**INTRODUCTION**
Rationale	**3**	Describe the rationale for the review in the context of existing knowledge.	Section 1 (Introduction). Comprehensive background on fasciolosis epidemiology, economic burden, pathogenesis, chemotherapy limitations, and the identified gap in comparative synthesis of vaccine antigens across ruminant hosts.
Objectives	**4**	Provide an explicit statement of the objective(s) or question(s) the review addresses.	End of Section 1 and Abstract. Explicit objectives: identify principal antigen candidates, describe study characteristics and outcome measures, and assess reporting quality of vaccines against F. hepatica and F. gigantica in cattle, sheep and goats.
**METHODS**	**METHODS**	**METHODS**	**METHODS**
Eligibility criteria	**5**	Specify the inclusion and exclusion criteria for the review and how studies were grouped for the syntheses.	Section 2.1 and Chart 1. Inclusion: original English articles evaluating vaccine efficacy against F. hepatica or F. gigantica in cattle/buffaloes/goats/sheep with immune-response outcomes and appropriate controls. Exclusion: other species, editorials/reviews/conference abstracts, F. magna. Studies grouped by parasite species.
Information sources	**6**	Specify all databases, registers, websites, organisations, reference lists and other sources searched or consulted to identify studies. Specify the date when each source was last searched or consulted.	Section 2.1. MEDLINE (PubMed), Google Scholar, Cochrane Library (via VHL). Initial search June 2024; last updated and searched 10 May 2026. Reference lists of retrieved publications hand-searched.
Search strategy	**7**	Present the full search strategies for all databases, registers and websites, including any filters and limits used.	Chart 1. Full Boolean search strings provided for PubMed, Google Scholar and Cochrane Library.
Selection process	**8**	Specify the methods used to decide whether a study met the inclusion criteria of the review, including how many reviewers screened each record and each report retrieved, whether they worked independently, and if applicable, details of automation tools used in the process.	Section 2.1. Two independent reviewers (GD, LA) screened titles, abstracts and full texts using the Rayyan platform. Disagreements resolved by consensus with a third researcher.
Data collection process	**9**	Specify the methods used to collect data from reports, including how many reviewers collected data from each report, whether they worked independently, any processes for obtaining or confirming data from study investigators, and if applicable, details of automation tools used in the process.	Section 2.2. Independent extraction by two reviewers (GD, LA) following a fixed protocol. Disagreements resolved by a third researcher. Authors contacted for missing information when necessary.
Data items	**10a**	List and define all outcomes for which data were sought. Specify whether all results that were compatible with each outcome domain in each study were sought (e.g. for all measures, time points, analyses), and if not, the methods used to decide which results to collect.	Section 2.2 and Results 3.2. Outcomes sought: antigen identity and dose, adjuvant, vaccination schedule, challenge dose, serological (ELISA) and parasitological (fluke burden, faecal egg count) responses, liver pathology, weight gain and related efficacy measures. All compatible results were extracted.
Data items	**10b**	List and define all other variables for which data were sought (e.g. participant and intervention characteristics, funding sources). Describe any assumptions made about any missing or unclear information.	Section 2.2. Additional variables: host species, geographic location, ethics-committee approval, journal impact factor (2025 values), sample-size justification and scientific background justification. Missing data recorded as “not reported”.
Study risk of bias assessment	**11**	Specify the methods used to assess risk of bias in the included studies, including details of the tool(s) used, how many reviewers assessed each study and whether they worked independently, and if applicable, details of automation tools used in the process.	Section 2.3. Reporting quality assessed independently by two reviewers using an adapted CONSORT 2010 checklist (piloted beforehand). Disagreements resolved by a third reviewer. Formal risk-of-bias tools (Cochrane RoB 2 / ROBINS-I) were outside

			the declared scope of this reporting-quality-focused review.
Effect measures	**12**	Specify for each outcome the effect measure(s) (e.g. risk ratio, mean difference) used in the synthesis or presentation of results.	Not applicable. The review is qualitative/descriptive and does not perform quantitative synthesis of efficacy outcomes (explicitly stated in the Discussion).
Synthesis methods	**13a**	Describe the processes used to decide which studies were eligible for each synthesis (e.g. tabulating the study intervention characteristics and comparing against the planned groups for each synthesis (item #5)).	Sections 2.1 and 3.1–3.2. Studies stratified a priori by parasite species (F. hepatica vs F. gigantica). Eligibility determined by full-text assessment against Chart 1 criteria.
Synthesis methods	**13b**	Describe any methods required to prepare the data for presentation or synthesis, such as handling of missing summary statistics, or data conversions.	Not applicable (no quantitative synthesis). Missing data simply recorded as “not reported”.
Synthesis methods	**13c**	Describe any methods used to tabulate or visually display results of individual studies and syntheses.	Results 3.2–3.6 and Supplementary Tables 01–03. Narrative synthesis supported by structured tables of antigens, study characteristics, geographic distribution (Figure 2) and CONSORT compliance scores.
Synthesis methods	**13d**	Describe any methods used to synthesize results and provide a rationale for the choice(s). If meta-analysis was performed, describe the model(s), method(s) to identify the presence and extent of statistical heterogeneity, and software package(s) used.	Narrative qualitative synthesis only. Rationale: substantial clinical and methodological heterogeneity in antigens, adjuvants, hosts and outcome measures precluded meta-analysis. The review explicitly does not synthesise efficacy outcomes.
Synthesis methods	**13e**	Describe any methods used to explore possible causes of heterogeneity among study results (e.g. subgroup analysis, meta-regression).	Not applicable (no quantitative synthesis). Heterogeneity is described narratively by host species, antigen type and geographic location.
Synthesis methods	**13f**	Describe any sensitivity analyses conducted to assess robustness of the synthesized results.	Not applicable (no quantitative synthesis).
Reporting bias assessment	**14**	Describe any methods used to assess risk of bias due to missing results in a synthesis (arising from reporting biases).	Section 2.2. Restriction to full-text English articles only (abstracts, conference proceedings and unpublished data excluded), introducing potential publication bias, which is acknowledged.
Certainty assessment	**15**	Describe any methods used to assess certainty (or confidence) in the body of evidence for an outcome.	Not performed. The review focuses on identification of antigen candidates and reporting quality rather than grading certainty of efficacy evidence.
**RESULTS**	**RESULTS**	**RESULTS**	**RESULTS**
Study selection	**16a**	Describe the results of the search and selection process, from the number of records identified in the search to the number of studies included in the review, ideally using a flow diagram.	Section 3.1 and Figure 1 (PRISMA flow diagram). 751 (PubMed) + 5 000 (Google Scholar) + 0 (Cochrane) records; after title screening 5 599 excluded, leaving 152 potentially eligible; after duplicate removal, abstract and full-text screening, 44 studies included (37 F. hepatica, 7 F. gigantica).
Study selection	**16b**	Cite studies that might appear to meet the inclusion criteria, but which were excluded, and explain why they were excluded.	Section 3.1. Transparent exclusion counts: duplicates (n=20), reviews (n=10), non-English (n=5), immune-response-only studies (n=27), missing humoral data (n=16), non-target species (n=8 + 4). Reasons fully reported; individual citations of excluded studies omitted because of volume (accepted practice).
Study characteristics	**17**	Cite each included study and present its characteristics.	Sections 3.2–3.4 and Supplementary Tables 01 (F. gigantica) and 02 (F. hepatica). All 44 studies are cited and characterised by antigen, host, adjuvant, doses, outcome measures, location and journal impact factor.
Risk of bias in studies	**18**	Present assessments of risk of bias for each included study.	Section 3.6 and Supplementary Table 03. Reporting-quality scores (CONSORT items fulfilled) presented for every included study. Highest compliance: 35.1 % (F. gigantica) and 24.3 % (F. hepatica).
Results of individual studies	**19**	For all outcomes, present, for each study: (a) summary statistics for each group (where appropriate) and (b) an effect estimate and its precision (e.g. confidence/credible interval), ideally using structured tables or plots.	Not applicable for quantitative effect estimates (review scope is descriptive). Key characteristics and qualitative findings of individual studies are presented narratively in 3.2.1–3.2.2 and in the Supplementary Tables.
Results of syntheses	**20a**	For each synthesis, briefly summarise the characteristics and risk of bias among contributing studies.	Sections 3.2–3.6. Narrative summaries of antigen frequency, adjuvant use, outcome measures, geographic distribution, ethics

			reporting, journal impact factor, sample-size justification and CONSORT compliance provided separately for each parasite species.
Results of syntheses	**20b**	Present results of all statistical syntheses conducted. If meta-analysis was done, present for each the summary estimate and its precision (e.g. confidence/credible interval) and measures of statistical heterogeneity. If comparing groups, describe the direction of the effect.	Not applicable – no statistical synthesis or meta-analysis performed.
Results of syntheses	**20c**	Present results of all investigations of possible causes of heterogeneity among study results.	Not applicable.
Results of syntheses	**20d**	Present results of all sensitivity analyses conducted to assess the robustness of the synthesized results.	Not applicable.
Reporting biases	**21**	Present assessments of risk of bias due to missing results (arising from reporting biases) for each synthesis assessed.	Acknowledged in Methods 2.2 and Discussion: restriction to full-text English articles may introduce publication bias; no formal statistical assessment performed.
Certainty of evidence	**22**	Present assessments of certainty (or confidence) in the body of evidence for each outcome assessed.	Not performed (outside the defined scope of the review).
**DISCUSSION**	**DISCUSSION**	**DISCUSSION**	**DISCUSSION**
Discussion	**23a**	Provide a general interpretation of the results in the context of other evidence.	Section 4. Interprets predominance of rFhCL1 and rFgFABP, protocol heterogeneity, poor CONSORT compliance, and the promise of multivalent and phage-display approaches.
Discussion	**23b**	Discuss any limitations of the evidence included in the review.	Sections 4 and 4.1. Limitations of included evidence: suboptimal reporting quality, absence of sample-size justification, incomplete ethics and location reporting, heterogeneous pre-challenge screening, and lack of dual-species comparative trials.
Discussion	**23c**	Discuss any limitations of the review processes used.	Section 4.1. Limitations of the review process: equal weighting of CONSORT items; focus on reporting quality rather than methodological risk of bias; restriction to English full-text articles.
Discussion	**23d**	Discuss implications of the results for practice, policy, and future research.	Section 4 (final paragraphs) and Section 5 (Conclusion). Concrete recommendations: standardised protocols, multivalent formulations, head-to-head comparative trials, mandatory CONSORT checklist submission by journals, and prioritisation of adjuvant optimisation.
**OTHER INFORMATION**	**OTHER INFORMATION**	**OTHER INFORMATION**	**OTHER INFORMATION**
Registration and protocol	**24a**	Provide registration information for the review, including register name and registration number, or state that the review was not registered.	Section 2.1. Protocol registered retrospectively in the Open Science Framework (OSF) on 8 July 2026 (https://osf.io/mwgvf; DOI: 10.17605/OSF.IO/39WHS).
Registration and protocol	**24b**	Indicate where the review protocol can be accessed, or state that a protocol was not prepared.	Section 2.1. Protocol publicly accessible via the OSF link and DOI provided.
Registration and protocol	**24c**	Describe and explain any amendments to information provided at registration or in the protocol.	Not explicitly described in the current manuscript draft. A brief statement should be added if any amendments occurred after registration.
Support	**25**	Describe sources of financial or non-financial support for the review, and the role of the funders or sponsors in the review.	Not reported in the current draft. A Funding / Support statement must be added before journal submission.
Competing interests	**26**	Declare any competing interests of review authors.	Not reported in the current draft. A Competing Interests / Conflict of Interest declaration must be added before journal submission.
Availability of data, code and other materials	**27**	Report which of the following are publicly available and where they can be found: template data collection forms; data extracted from included studies; data used for all analyses; analytic code; any other materials used in the review.	Supplementary Tables 01–03 contain the extracted data. Authors are encouraged to deposit the full data-extraction spreadsheet (and any code) on the existing OSF project page and to state this availability explicitly.

Page MJ, McKenzie JE, Bossuyt PM, et al. The PRISMA 2020 statement: an updated guideline for reporting systematic reviews. BMJ 2021;372:n71. doi: 10.1136/bmj.n71. Licensed under CC BY 4.0.
